# Thermal Positioning Error Modeling of Servo Axis Based on Empirical Modeling Method

**DOI:** 10.3390/mi12020201

**Published:** 2021-02-15

**Authors:** Yang Li, Hexuan Shi, Shijun Ji, Fusheng Liang

**Affiliations:** 1 School of Mechanic Engineering, Northeast Electric Power University, Jilin 132012, China; liyang891209@126.com (Y.L.); shihx0203@163.com (H.S.); 2 School of Mechanical and Aerospace Engineering, Jilin University, Changchun 130025, China; jishijun97@126.com; 3 School of Mechanical & Materials Engineering, University College Dublin, Dublin 4 D04 V1W8, Ireland

**Keywords:** thermal error modeling, fuzzy clustering analysis, principal component regression, temperature-sensitivity points, CNC machine tools

## Abstract

In order to investigate the thermal effect of a servo axis’ positioning error on the accuracy of machine tools, an empirical modeling method was proposed, which considers both the geometric and thermal positioning error. Through the analysis of the characteristics of the positioning error curves, the initial geometric positioning error was modeled with polynomial fitting, while the thermal positioning error was built with an empirical modeling method. Empirical modeling maps the relationship between the temperature points and thermal error directly, where the multi-collinearity among the temperature variables exists. Therefore, fuzzy clustering combined with principal component regression (PCR) is applied to the thermal error modeling. The PCR model can preserve information from raw variables and eliminate the effect of multi-collinearity on the error model to a certain degree. The advantages of this modeling method are its high-precision and strong robustness. Experiments were conducted on a three-axis machine tool. A criterion was also proposed to select the temperature-sensitivity points. The fitting accuracy of the comprehensive error modeling could reach about 89%, and the prediction accuracy could reach about 86%. The proposed modeling method was proven to be effective and accurate enough to predict the positioning error at any time during the machine tool operation.

## 1. Introduction

With the development of the machine manufacturing industry, the demand for high-precision machine tools is increasing. Not only a better accuracy, but also a higher removal rate is provided by high-precision machine tools than that of traditional machine tools. High-speed machining technology can provide high spindle revolution, high axial feed rate, and a high-CPU (Central Processing Unit) processing speed. Machining efficiency can be improved greatly, and the machining time can be reduced significantly. However, the more heat is generated by the high-speed machining technology, and the more the accuracy of the machine tools is damaged. Thermally induced error is demonstrated as one of the greatest contributors to the accuracy of the high-precision and ultra-precision machine tools. A thermally induced error can account for 40–70% of the total errors [1,2,3], which must be reduced to keep the accuracy of the machine tools during processing.

Generally speaking, there are two ways to reduce the thermal error: error avoidance and error compensation [4]. In the error avoidance, the thermally symmetric design of machine structure, the separation of heat sources, rearrangement of machine tool structures, the improvement of the rigidity of machine tool structure, and materials with low thermal expansion coefficient, an air-cooling system with a hollow ball screw, room temperature-controlled workshop, etc. are the common methods to reduce the thermal error [5,6]. The basic accuracy of machine tools can be ensured in this way, but the costs dramatically increase with the increase in accuracy grade. In contrast, error compensation is a cost-efficient way to enhance the accuracy of machine tools [7]. A thermal error is predicted by the error model, and then compensated by the software [8]. The main steps of error compensation are: (1) the relationship between the errors and the location is established considering temperatures; (2) the compensation signals based on the error model sent to the CNC (Computer Numerical Control) controllers; (3) the tool and the workpiece have relative motions in the opposite direction to the predicted errors.

Thermal error measurement, error modeling, and error compensation have been the focus of many significant studies in recent years. Two popular methods of thermal error modeling are theoretical modeling and empirical modeling. In the theoretical modeling, through the calculations of heat generations and convective heat transfer coefficients of different components in a machine tool, the temperature distribution can be obtained, and the deformation can also be gained according to thermo-elasticity. Both the analytic method and the numerical method could effectively solve the problems of establishing differential equations. The finite element method with the help of mathematical software is the most common method. However, the accurate boundary conditions and the heat transfer characteristics of theoretical modeling are in need of theoretical modeling, which are difficult to clearly identify. A three-dimensional FEA (Finite Element Analysis) model was proposed to conduct a transient thermal structure interactive analysis of a high-speed spindle by Ma et al. [9]. Shi et al. [10] investigated the effect of thermal expansion on the ball screw feed drive system of a precision boring machine tool, and the theoretical model for thermally induced error along with heat generation characteristics was established. Zhang et al. [11] used the finite element method to predict the temperature field of a high-speed and high-precision motorized spindle under different working conditions.

Empirical modeling is different from theoretical modeling, where the relationship between the thermal errors and the temperature measurements was mapped by the data-driven models such as the neural network [12,13], gray model [14], support vector model [15], and time series model [16]. A thermal error model with the four key temperature points was proposed by Guo et al. [17] using an ant colony algorithm-based back propagation neural network (ACO-BPN). Wang et al. [18] proposed a compound error model by Newton interpolation for the geometric and thermal errors of a milling center. A comprehensive compensation model was established through the decomposition of the geometric error and thermal error components by Li et al. [19], where the thermal effects caused by internal and external heat sources were modeled separately. Xiang et al. [20] presented a strategy to build an error model of an NC (Numerical Control) lathe considering both thermal and load effects. An offline compensation technique modifying the NC G-codes for positional, geometrical, and thermally induced errors of machine tools was presented by Eskandari et al. [21].

In this paper, an empirical modeling was applied to build the thermal error model. The core idea of empirical modeling was to map the relationship between the thermal errors and the temperature measurements. However, if the multi-collinearity among the temperature variables existed, the accuracy and the robustness of the thermal error modeling would be directly affected. Therefore, the highly correlated variables must be screened out. Various approaches were presented to eliminate the influence of the multi-collinearity among the variables. The gray correlation theory [22] and the clustering [23,24,25,26] are the commonly used methods to optimize the temperature variables. The fuzzy clustering analysis, gray correlation, stepwise regression, and determination coefficient were combined to select temperature-sensitive points by Miao et al. [27].

The establishment of the thermal error model has two requirements. One is that the model has a high enough accuracy, and the other one is that the model has a strong enough robustness. Therefore, a thermal error modeling method considering these two requirements is presented in this paper.

The multi-collinearity among independent variables needs to be removed before establishing the thermal error model. In the traditional methods, after the classification, the representative variable in each group is selected, and the variables finally chosen to build the error model from these representative variables are determined by the least degree of multi-collinearity. The benefits of the traditional method are that the multi-collinearity can be furthest reduced, and the predicted robustness can be guaranteed.

Generally, the stronger the correlation between input variables and the thermal error is, the more accurate the precision of the model is. However, some variables which have low correlation with respect to the thermal error are picked out by these methods, which may decrease the accuracy and robustness of the thermal error model. Therefore, in this paper, the selection method of variables is different from the traditional ones. After the representative variables are picked out, the fitting accuracy of the error model should be considered first. The variables for building the thermal error model are selected based on sample determination coefficient and the significance of the regression equation, which gives priority to the fitting precision of the error model. However, there may be still multi-collinearity among the input variables selected by this method. Principal component regression is then applied to build the thermal error model. Multi-collinearity among the input variables can be further reduced. The robustness and prediction accuracy of the error model can be enhanced.

The rest of this paper is arranged as follows: in Section 2, the modeling of principal component regression is established. Section 3 deals with the selection of temperature measuring points based on fuzzy clustering analysis. A three-axis machine tool is taken as an example to verify the proposed method in Section 4, and some conclusions are presented in Section 5.

## 2. Modeling of Thermal Positioning Error

**Figure 1:**
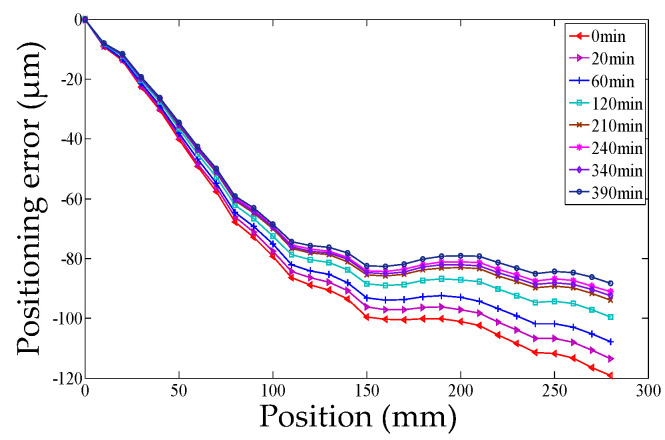
*Y* axis positioning error under different temperature conditions.

It is well known that the positioning error of the servo axis is not only related to the position coordinate but also affected by the temperature field as shown in Figure 1.

### 2.1. The Characteristic Thermal Positioning Error

Geometric error and thermal error mostly contribute to the positioning error. The other error sources such as loads, dynamic forces, motion control, and the control software, account for a small portion of the positioning error. Therefore, only the geometric error term and the thermal error term are taken into account when the model of the positioning error is built [19,28,29,30]. The geometric error term together with the thermal error term can be easily measured by a laser interferometer.

The equation of the positioning error of the *P* axis (*P* = *X*, *Y*, *Z*) is expressed as

(1) $$\delta_{PP}(P,T)=\delta_{G}(P)+\delta_{T}(P)+\varepsilon$$

where $\delta_{PP}(P,T)$ is the total positioning error of the *P* axis (*P* = *X*, *Y*, *Z*); $\delta_{G}(P)$ is the geometric error term measured at the cold state, which is only related to the location of the *P* axis; $\delta_{T}(P)$ is the thermally induced positioning error which is related to the machine temperature field and the location of the *P* axis; *ε* is other error sources of the *P* axis positioning error.

The initial positioning error $\delta_{G}(P)$ can be fitted by polynomial as expressed in Equation (2), and the thermally induced positioning error $\delta_{T}(P)$ is shown in Equation (3):

(2) $$\delta_{G}(P)=\sum_{i=0}^{n}\alpha_{i}p^{i}$$

(3) $$\delta_{T}(P)=(k_{i}-k_{0})p$$

where $\alpha_{i}$ is the coefficient of the polynomial;p is the nominal position of the *P* axis; $k_{i}$ is the slope of each positioning error curve;$k_{0}$ is the slope of the initial positioning error curve.

The thermally induced positioning error $\delta_{T}(P)$ can be calculated as follows:

(1) Calculate the slope ($k_{i}$) of each error curve. According to the first-order polynomial fitting, a series of slopes can be obtained.
(2) Map relationship between the slopes and the pivotal temperatures.

The slope $k_{i}$ is a linear function of the pivotal temperature variables which are selected by fuzzy clustering analysis. The details of selection are described in the next section. Usually, the relationship between the slopes and the pivotal temperature variables can be built by multiple linear regression (MLR):

(4) $$k_{i}=\beta_{0}+\beta_{1}T_{1}+\beta_{2}T_{2}+\cdots +\beta_{n}T_{n}+\varepsilon$$

### 2.2. Principal Component Regression

When the thermal error model is built by the MLR, multi-collinearity among variables exists, and the prediction accuracy and robustness of the error model are degraded. Therefore, principal component analysis is presented to eliminate the influence of multi-collinearity among variables. PCR (principal component regression) is also one kind of mathematical statistics methods, which is usually applied to the regression analysis. Principal components are unrelated to each other and carry the most information among raw variables.

The process of PCR is mainly divided into five steps:

(1) Standardize the data;
(2) Calculate correlation matrix R of the standardized data;
(3) Calculate the principal components according to the correlation matrix;
(4) Select the principal components. Principal components are selected according to the scree plot and cumulative percentage which is usually bigger than 85%;
(5) Carry out the regression analysis with the selected principal components.

The slope $k_{i}$ is a linear function of the principal components instead of the raw temperature variables:

(5) k=βP

where $k=\left[\begin{array}{c} k_{0}\\ k_{1}\\ \vdots \\ k_{p} \end{array}\right]$, $\beta ={\left[\begin{array}{c} \beta_{0}\\ \beta_{1}\\ \vdots \\ \beta_{p} \end{array}\right]}^{T}$ are the regression coefficients; $P=\left[\begin{array}{c} 1\\ P_{1}\\ \vdots \\ P_{p} \end{array}\right]=\left[\begin{array}{c} 1\\ P\prime \end{array}\right]$ are the selected principal components:

(6) P′=αT

where $P\prime =\left[\begin{array}{c} P_{1}\\ P_{2}\\ \vdots \\ P_{p} \end{array}\right]$, $T=\left[\begin{array}{c} T_{1}\\ T_{2}\\ \vdots \\ T_{n} \end{array}\right]$, and $\alpha =\left[\begin{array}{cccc} \alpha_{11} & \alpha_{12} & \cdots & \alpha_{1n}\\ \alpha_{21} & \alpha_{22} & \cdots & \alpha_{2n}\\ \vdots & \vdots & \vdots & \vdots \\ \alpha_{p1} & \alpha_{p2} & \cdots & \alpha_{pn} \end{array}\right]$

## 3. Selection of Temperature Measuring Points

Thermal error modeling is a challenging task because the mechanism causing the machine tool deformations is so complex that thermal error cannot be accurately predicted. In order to improve the robustness and the prediction accuracy of the error model, the multi-collinearity among the temperature variables must be eliminated through selecting the representative temperature variables from many temperature sensors preliminarily installed on the machine tool. The essence of identifying the representative temperature variables is to conduct classification, and then select one variable from each group to represent this category. The clustering analysis is one of the most common tools for the classification.

### 3.1. Clustering Analysis

Fuzzy cluster analysis is widely used, which has unique advantages compared to the traditional ones. In this paper, the maximal tree method was chosen.

The maximal tree method is implemented through the following steps. Firstly, correlation analysis between every two temperature variables is made, and the fuzzy similarity matrix is obtained. Secondly, the maximal tree is built based on the prim method. Thirdly, clustering analysis is conducted to classify the whole temperature variables. Lastly, representative temperature variables are gained from each clustering based on the correlation coefficient between the temperature variables and the thermal error. The permutation and combination of these representative temperature variables are regarded as input variables of the error model.

### 3.2. Clustering Criterion

In the process of clustering, the criterion of selecting the optimal clustering result is very important. The criterion is a quantitative indicator determine the optimal clustering result from the whole clustering results. In this paper, the sample determination coefficient (*R*^2^) and the significance of the regression equation were taken as the criteria.

The sample determination coefficient is:

(7) $$R^{2}=\frac{SSR}{SST}=1-\frac{SSE}{SST}$$

$SST=\sum_{i=1}^{n}{\left(k_{i}-\bar{k}\right)}^{2}$, $SSR=\sum_{i=1}^{n}{\left({\hat{k}}_{i}-\bar{k}\right)}^{2}$, $SSE=\sum_{i=1}^{n}{\left(k_{i}-{\hat{k}}_{i}\right)}^{2}$, and SST=SSR+SSE. where $k_{i}$ is the measured value, ${\hat{k}}_{i}$ is the fitted value, and $\bar{k}=\sum_{i}^{n}k_{i}$.

*F* statistics is constructed:

(8) $$F=\frac{SSR/p}{SSR/\left(n-p-1\right)}$$

This obeys *F* distribution, whose degrees of freedom are *p* and *n*−*p*−1. When $F>F_{\alpha }(p,n-p-1)$, the regression equation is significant under the significance level which is usually equal to 0.05. On the contrary, the regression equation is not significant.

**Figure 2:**
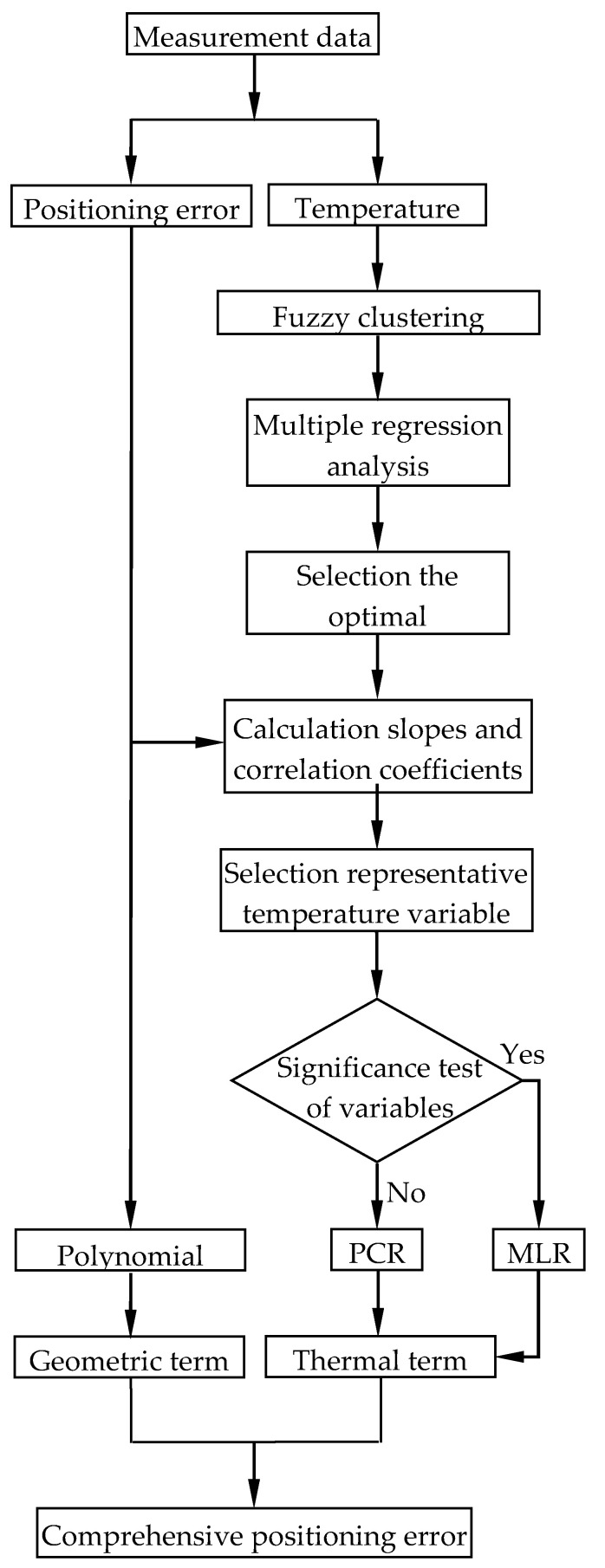
The flow chart of the thermal positioning error modeling.

The closer *R*^2^ is to 1 and the more significant the regression equation is, the better the optimal clustering result is. The flow chart of the thermal positioning error modeling is shown in Figure 2, where MLR is abbreviated as multiple linear regression.

## 4. Experiment and Verification

**Figure 3:**
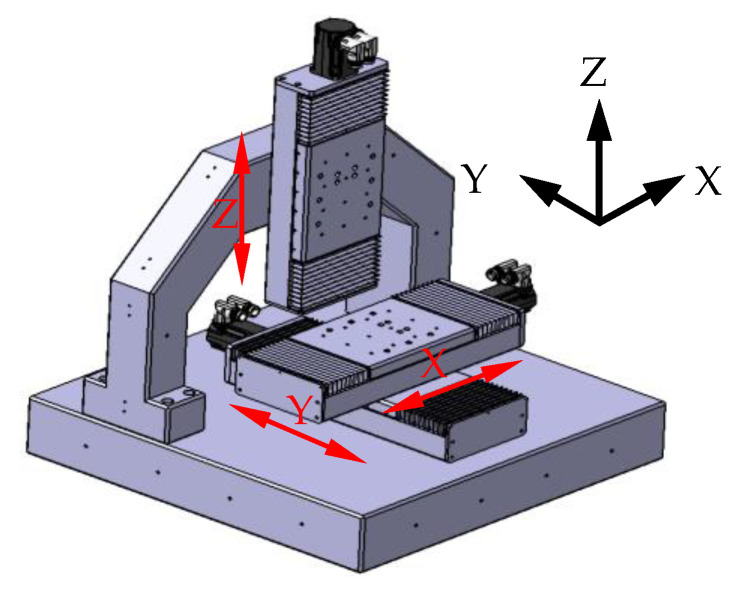
The structure of the 3-axis machine tool.

**Figure 4:**
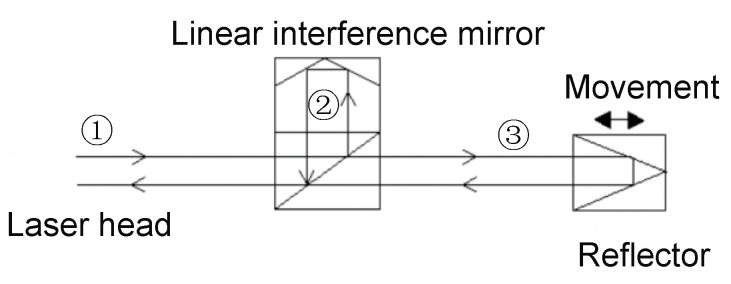
Measurement principle of the laser interferometer.

The experiments were conducted on a three-axis machine tool whose structure is shown in Figure 3. The positioning error of the *Y* axis was taken as an example to verify the thermal positioning error modeling, the modeling processes and experiments of the *X* axis and the *Z* axis were similar to the *Y* axis. A laser interferometer (Renishaw XL-80) was used to measure the positioning error, and the principle of measurement was shown in Figure 4. Measurement was based on the principle of light interference. A laser beam ① was emitted from the laser head, which was divided into two beams of light ② and ③ through the interference mirror. Beam ② directly returns to the receiving point of the laser head through the interference mirror, and beam ③ reflects to the receiving point by the reflector. The displacement of the reflector can be obtained according to the number of pulses.

**Figure 5:**
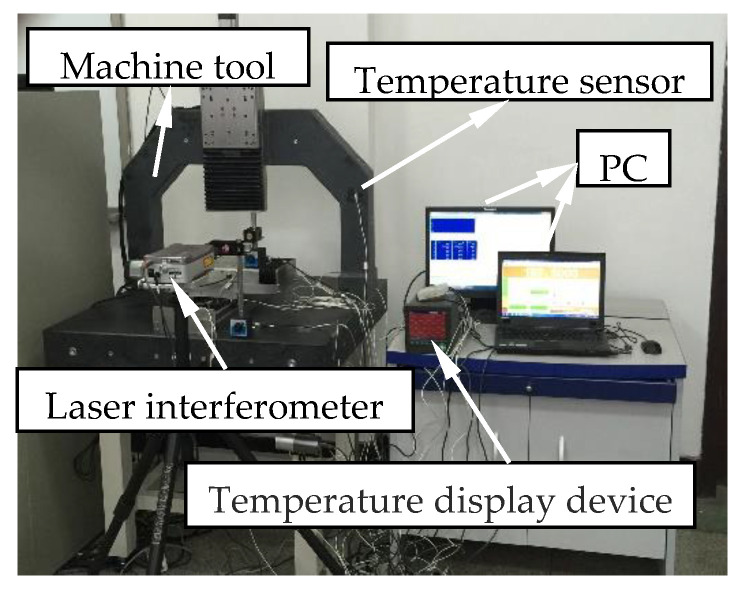
Measurement set-up.

**Figure 6:**
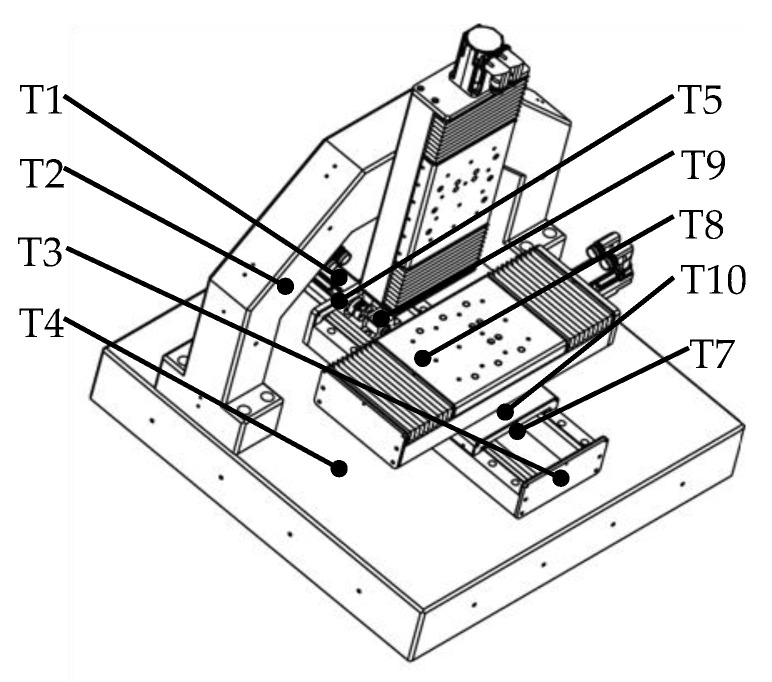
The arrangement of temperature sensors.

The distribution of temperature was detected by the PT100 temperature sensors, where the Pt 100 temperature sensors with two wires were used. The scene of the measurement can be seen in Figure 5. Considering the structure of the machine tool and the actual operating conditions, 11 temperature sensors were preliminarily installed on the parts of the machine tool, such as the bed, screw, and similar others. The arrangement of the sensors is shown in Figure 6 where T means the temperature sensor. Here, T6 and T11 are not included. The installation locations of the temperature sensors are explained in Table 1.

### 4.1. Thermal Positioning Error Measurement and Modeling

The starting point of the positioning error measurement was set to be the machine reference origin of the *Y* axis, and the positioning error was bi-directionally measured every 10 mm in the whole stroke range of 280 mm. Firstly, the geometric positioning error was measured when the machine tool was initially switched on. Then, the machine tool is cooled down to the initial condition which is the same as the first measurement, and the positioning error was measured again. This process was repeated four times, and the geometric positioning error was calculated by averaging the obtained five groups of data. The machine tool was warmed up by moving the *Y* axis slide all along its stroke with a feed rate of 20 mm/s and an acceleration of 10 mm/s^2^. However, the process of the positioning error measurement in the heating process was different from that of the geometric positioning error. When the machine tool began to be heated, the thermal state changed dramatically. Even the movement of the *Y* axis for measurement would make the thermal state change greatly. Therefore, the measurement of the positioning error was repeated only twice at the beginning of the heating process. With the development of the heating process, the change in thermal state was gradually slow. At this stage, the positioning error was measured three times. Finally, the machine tool reached the thermal equilibrium state which changed very little, and the positioning error was measured five times. Meanwhile, the temperatures were measured at an interval of 10 min until the ball screw system reached a thermal equilibrium state. As shown in Figure 1, the measurements of the positioning error were synchronized with the temperature measurements after the machine tool had been warmed up for 0, 20, 60, 120, 210, 240, 340, and 390 min, respectively (represented by the numbers 0, 1, 2, 3, 4, 5, 6, and 7).

Modeling for the thermal positioning error could be carried out. Firstly, the initial positioning error was modeled with polynomial fitting, for which the expression is:

(9) $$\begin{array}{ll} \delta_{G}(Y)= & 6.8499\times 10^{-10}y^{5}-5.5207\times 10^{-7}y^{4}+1.5173\times 10^{-4}y^{3}-0.0144y^{2}\\ & -0.3732y-1.6437 \end{array}$$

Secondly, the slope of each trend line was calculated, which is given in Table 2. The thermally induced positioning error is:

(10) $$\delta_{T}(Y)=(k_{i}-k_{0})y=\Delta ky$$

The slope $k_{i}$ is a linear function of the key temperature variables which are selected in the following.

### 4.2. Selection of Key Temperature Variables

**Figure 7:**
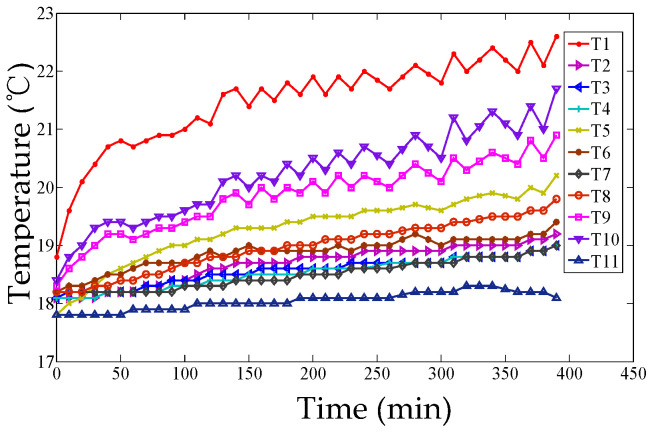
Temperatures of 11 temperature sensors.

Eleven sensors (PT100) were preliminarily installed on the machine tool, and the temperature curves of 11 temperature sensors were plotted at intervals of 10 min and are described in Figure 7. It can be seen that the temperatures are rising until the ball screw system reached a thermal equilibrium state.

The key temperature variables were selected by fuzzy clustering analysis. The steps of the maximal tree method were implemented as follows:

(1) The correlation coefficient between every two temperature variables is calculated:

(11) $$r_{ij}=\frac{\sum_{k=1}^{m}\left(x_{ik}-\bar{x_{i}}\right)\left(x_{jk}-\bar{x_{j}}\right)}{\sqrt{{\sum_{k=1}^{m}\left(x_{ik}-\bar{x_{i}}\right)}^{2}}\sqrt{{\sum_{k=1}^{m}\left(x_{jk}-\bar{x_{j}}\right)}^{2}}}$$

Then, all the correlation coefficients that form the fuzzy similarity matrix are shown in Equation (12):

(12) $$R=\left[\begin{array}{ccccccccccc} 1 & 0.91291 & 0.8972 & 0.88765 & 0.97532 & 0.95647 & 0.81976 & 0.908 & 0.97891 & 0.95868 & 0.86397\\ & 1 & 0.98677 & 0.98366 & 0.96459 & 0.94869 & 0.93909 & 0.9865 & 0.9602 & 0.96513 & 0.94943\\ & & 1 & 0.98309 & 0.95065 & 0.93572 & 0.94915 & 0.98747 & 0.95176 & 0.96321 & 0.93318\\ & & & 1 & 0.94542 & 0.92853 & 0.96927 & 0.9921 & 0.9519 & 0.96418 & 0.94085\\ & & & & 1 & 0.98288 & 0.88303 & 0.96299 & 0.98226 & 0.96855 & 0.91988\\ & & & & & 1 & 0.86006 & 0.94684 & 0.96524 & 0.94873 & 0.89664\\ & & & & & & 1 & 0.96475 & 0.90592 & 0.93318 & 0.91032\\ & & & & & & & 1 & 0.96373 & 0.97348 & 0.94578\\ & & & & & & & & 1 & 0.98947 & 0.90734\\ & & & & & & & & & 1 & 0.91689\\ & & & & & & & & & & 1 \end{array}\right]$$

(2) The maximal tree is built based on the prim method. First, Variable 1 is taken, and the maximum correlation coefficient relative to Variable 1 is determined from Variable 2 to Variable 11. Here, $r_{1,9}=0.97891$ is maximum, and the tree is drawn as


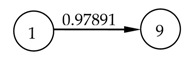


Then, in the rest of variables, the maximum correlation coefficients relative to Variables 1 and 9 are determined, respectively—which are $r_{1,5}=0.97532$ and $r_{9,10}=0.98947$. The bigger one remains and the tree is achieved.


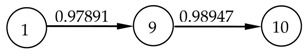


**Figure 8:**
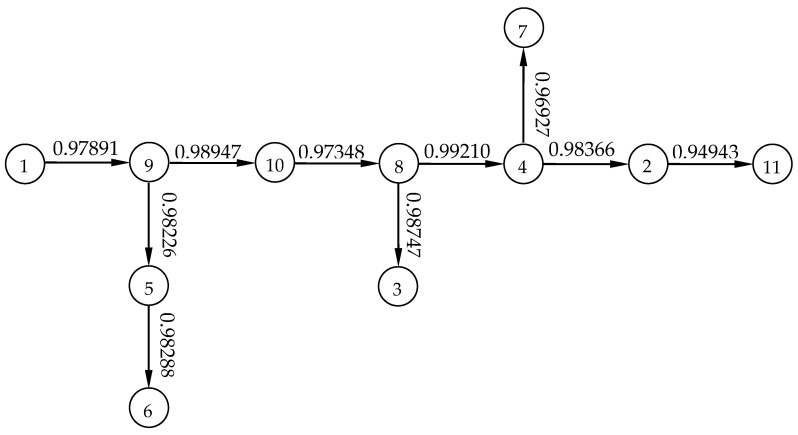
The maximal tree of the clustering analysis of the maximal tree method.

In a similar way, finally, the maximal tree is built as shown in Figure 8.

(3) Clustering analysis was conducted. When λ is in [0,1], the branches of the maximal tree whose correlation coefficients are less than λ are cut off, and a disconnected graph was obtained. The connected branches are the cluster results based on the threshold value λ. The cluster results can be seen in Table 3.

(4) The representative variable of each cluster is chosen. The temperature variable in each clustering who has the maximal correlation coefficient with respect to the slope $k_{i}$ is chosen as the representative temperature variable of this clustering. The correlation coefficients are calculated based on Equation (11), and the result is shown in Table 4. Based on the correlation coefficients, the combination of variables in each cluster result is shown in Table 5.

(5) The input variables of the error model are selected. When the optimal input variables are selected, the fitting accuracy of the error model should be considered first. According to the sample determination coefficient and the significance of the regression equation, the selection results are obtained in Table 6, Table 7 and Table 8. When the number of variables is 5, the sample determination coefficient is nearly equal to 1, and the regression equation is significant. While the number of variables is less than five, the sample determination coefficient is smaller than that of five variables. If the number of variables were 6, although the sample determination coefficient is equal to 1, the regression equation could be less significant than that of five variables. When the number of variables is bigger than six, the tolerance is exceeded, and some variables have to be excluded. Therefore, the best number of input variables is five, and they are $T_{1},T_{2},T_{5},T_{7},T_{11}$—as shown in Table 5.

The relationship between the slopes and five input variables is established by multiple regression analysis. The model summary and coefficients of the regression equation are shown in Table 9 and Table 10. Although the regression equation is significant, not all the variables pass the significance test. This means that the multi-collinearity exists among input variables. Therefore, a thermal error modeling method of principal component regression (PCR) algorithm is presented to eliminate the effect of multi-collinearity.

### 4.3. Principal Component Regression Modeling

Principal component analysis is one kind of mathematical statistics methods. PCR is applied to regression analysis with principal components which are unrelated to each other. Therefore, PCR can eliminate the influence of multi-collinearity among input variables. The construction of the principal component can be seen in Table 11.

**Figure 9:**
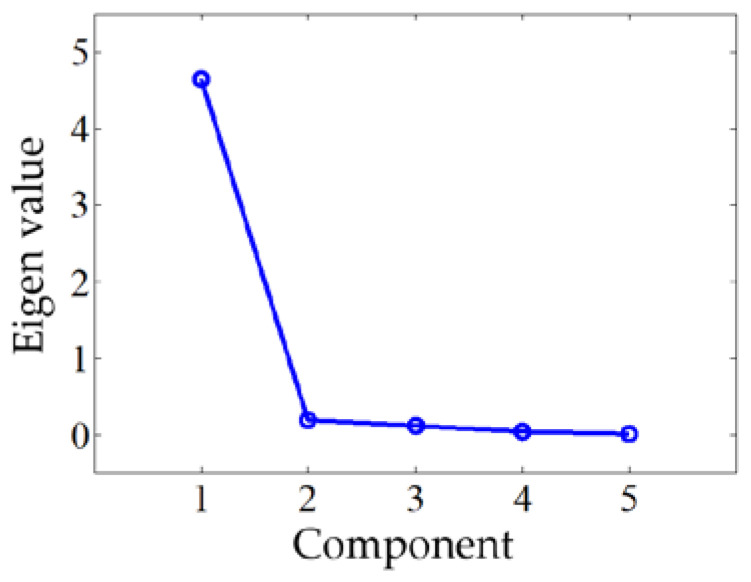
Scree plot of the PCR model.

According to the cumulative percentage bigger than 85% and scree plot shown in Figure 9, only the principal component 1 is selected as the input variable. The analysis results of PCR are given in Table 12, Table 13 and Table 14. The equation of PCR is:

(13) $$kp_{i}=-0.3243+0.0396C_{1i}$$

where kp is the slopes calculated by PCR, and $C_{1}$ is the principal component 1.

The relationship between principal component 1 and the five temperature variables is:

(14) $$C_{1i}=-50.6062+0.1631T_{1i}+0.4902T_{2i}+0.2512T_{5i}+0.6614T_{7i}+1.1666T_{11i}$$

The analysis results are shown in Table 15, Table 16 and Table 17. The equation of PCR with five temperature variables is:

(15) $$kp_{i}=0.0065T_{1i}+0.0194T_{2i}+0.0099T_{5i}+0.0262T_{7i}+0.0462T_{11i}-2.3283$$

The obtained model for the *Y* axis positioning error is expressed as follows:

(16) $$\begin{array}{ll} \delta P_{YY}(Y,T)= & 6.8499\times 10^{-10}y^{5}-5.5207\times 10^{-7}y^{4}+1.5173\times 10^{-4}y^{3}-0.0144y^{2}\\ & +(0.0065T_{1i}+0.0194T_{2i}+0.0099T_{5i}+0.0262T_{7i}+0.0461T_{11i}-2.3112)y\\ & -1.6437 \end{array}$$

Multiple linear regression (MLR) and artificial neural network (ANN) are also used to build the thermal error model to compare with the PCR. The input variables of the MLR and the ANN are the same as PCR.

The slope calculated by MLR is:

(17) $$km_{i}=-0.8782+0.0178T_{1}+0.0946T_{2}-0.0016T_{5i}-0.0608T_{7i}-0.0238T_{11i}$$

The *Y* axis thermal positioning error of MLR is expressed as follows:

(18) $$\begin{array}{ll} \delta M_{YY}(Y,T)= & 6.8499\times 10^{-10}y^{5}-5.5207\times 10^{-7}y^{4}+1.5173\times 10^{-4}y^{3}-0.0144y^{2}\\ & +(0.0178T_{1}+0.0946T_{2}-0.0016T_{5i}-0.0608T_{7i}-0.0238T_{11i}-0.8611)y\\ & -1.6437 \end{array}$$

### 4.4. Artificial Neural Network Modeling

**Figure 10:**
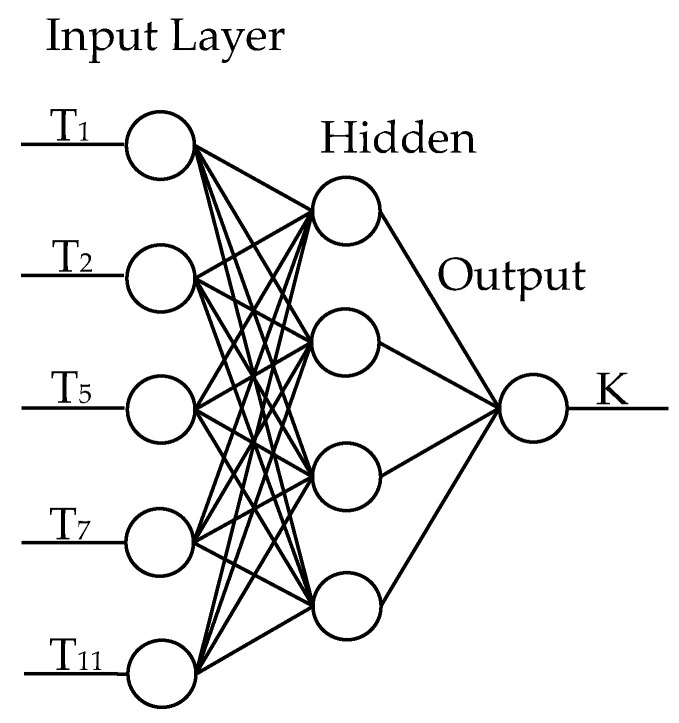
The structure of the neural network model.

Artificial neural network (ANN) is usually applied to nonlinear fitting. The neural network model is a network structure, which is composed of an input layer, hidden layers (one or two), and an output layer. For the neural network model shown in Figure 10, the node numbers of the input layer and output layer are determined by the input variables and output variables. The node numbers of the hidden layer can be selected by optimizing the structure of the neural network.

The neural network in this paper has only one hidden layer, as shown in Figure 10. The input layer has five nodes indicating that the model has five input variables, and the output layer has one node indicating that the model has one output variable. MSE (mean square error) and *R*^2^ for different number of nodes in the hidden layer are given in Table 18 and Table 19. It can be seen that the MSE and *R*^2^ of the hidden layer with four nodes, five nodes, and six nodes are almost the same, therefore the hidden layer with four nodes which has the least nodes of the hidden layer among three neural network model is selected.

### 4.5. The Modeling Results for the Y Axis Positioning Error

**Figure 11:**
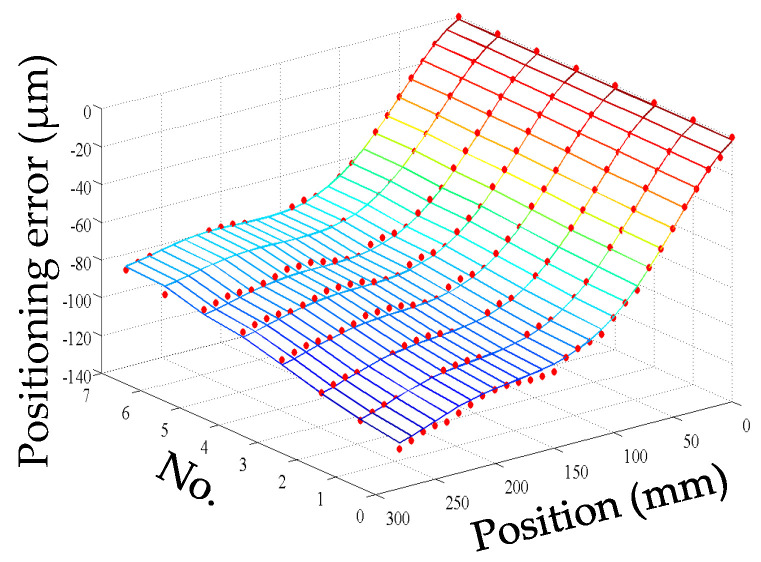
The fitting results of the PCR model (measured positioning error marked with dots) in experiment I.

**Figure 12:**
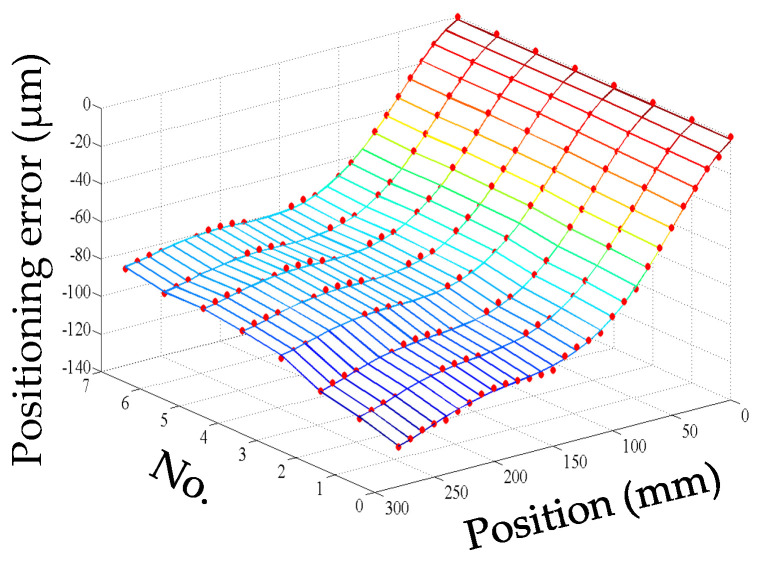
The fitting results of the MLR model (measured positioning error marked with dots) in experiment I.

**Figure 13:**
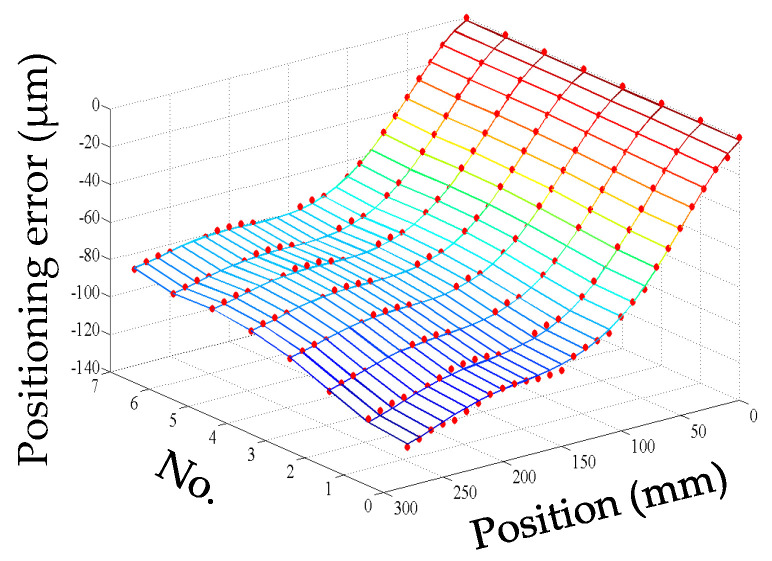
The fitting results of the artificial neural network (ANN) model (measured positioning error marked with dots) in experiment I.

**Figure 14:**
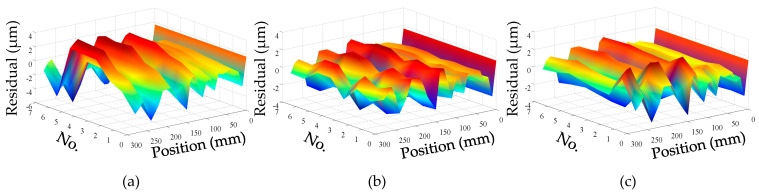
Residual error in experiment I: (**a**) residual error of PCR model; (**b**) residual error of MLR model; (**c**) residual error of ANN model.

For convenience, this experiment is abbreviated as experiment I. The modeling results of three models are shown in Figure 11, Figure 12 and Figure 13, and the residual error of three models can be seen in Figure 14. The performances of three models are calculated in Table 20 where the RMSE, MAXR, and MINR are short for root mean square error, maximal residual error, and minimum residual error, respectively.

Although the fitting accuracy of the PCR model is worse than that of the other two models, the fitting curves of the PCR model could match quite well with the actual measured values. This is because that the PCR model only contains the data of principal components which are less than the data carried by the MLR model or the ANN model. The performance of the MLR model is a little better than that of the ANN model. Even though the fitting accuracy of the PCR model is the worst, it can reach about 89% which is still very high.

### 4.6. Experimental Verification of the Positioning Error Modeling

**Figure 15:**
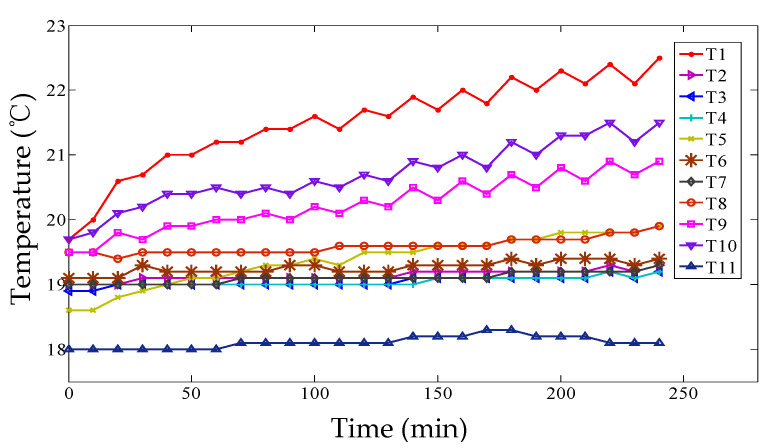
Temperatures of 11 temperature sensors of experiment II.

**Figure 16:**
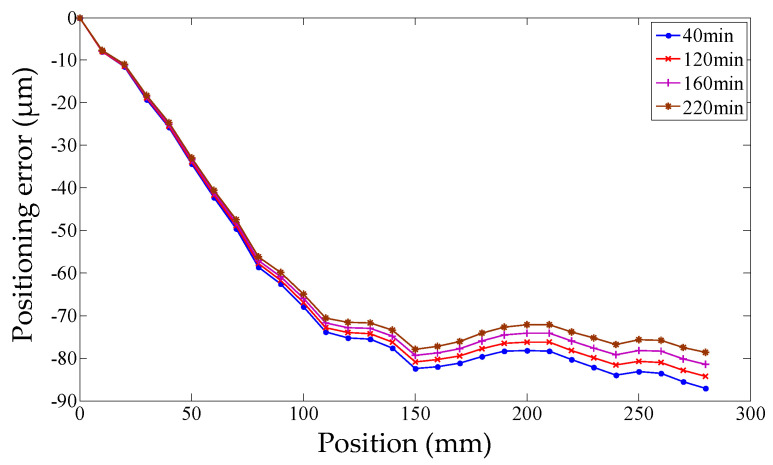
Positioning error of experiment II.

Subsequently, the robustness and predictive accuracy of the positioning error modeling were verified based on another set of experimental data (abbreviated as experiment II). The temperatures are plotted every 10 min in Figure 15, and the positioning error can be observed in Figure 16, where the feed rate is 15 mm/s and the acceleration is 8 mm/s^2^ to warm up the machine tool. The temperature of experiment II is different from experiment I. Environment temperatures of the room in two experiment are the main differentia. Experiment I was conducted in the daytime and experiment II was in the evening. Sunshine and central heating were also different.

**Figure 17:**
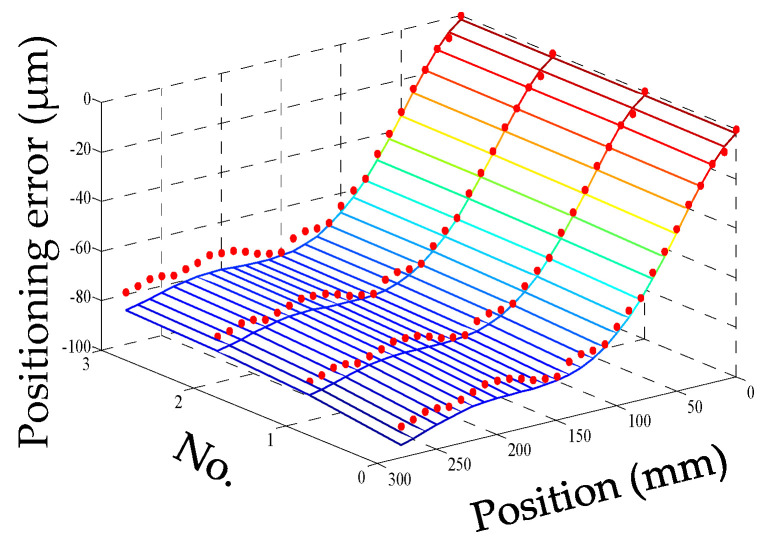
The prediction results of the PCR model (measured positioning error marked with dots) in experiment II.

**Figure 18:**
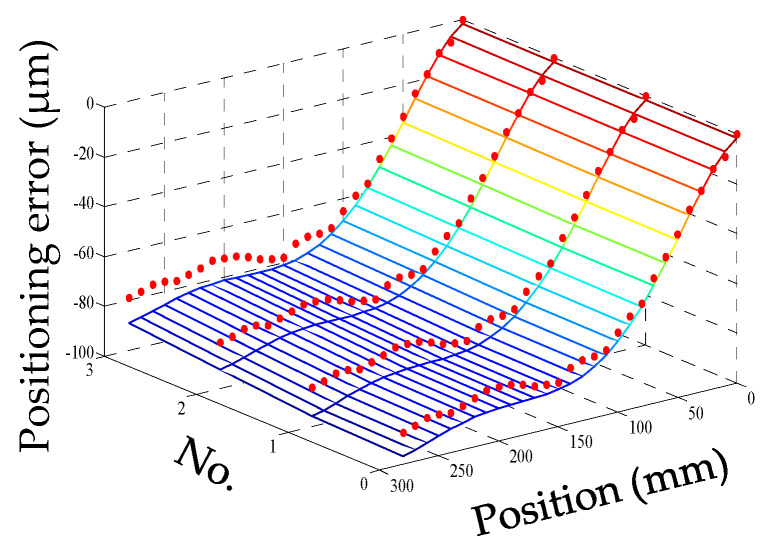
The prediction results of the MLR model (measured positioning error marked with dots) in experiment II.

**Figure 19:**
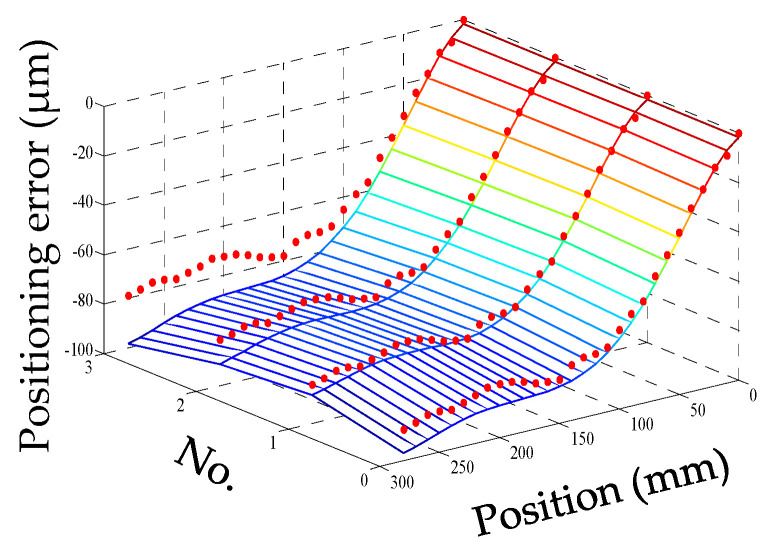
The prediction results of ANN model (measured positioning error marked with dots) in experiment II.

**Figure 20:**
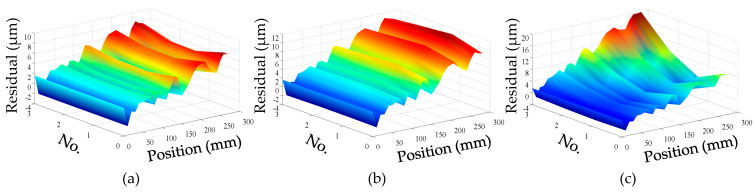
Residual error in experiment II: (**a**) residual error of the PCR model; (**b**) residual error of the MLR model; and (**c**) residual error of the ANN model.

**Figure 21:**
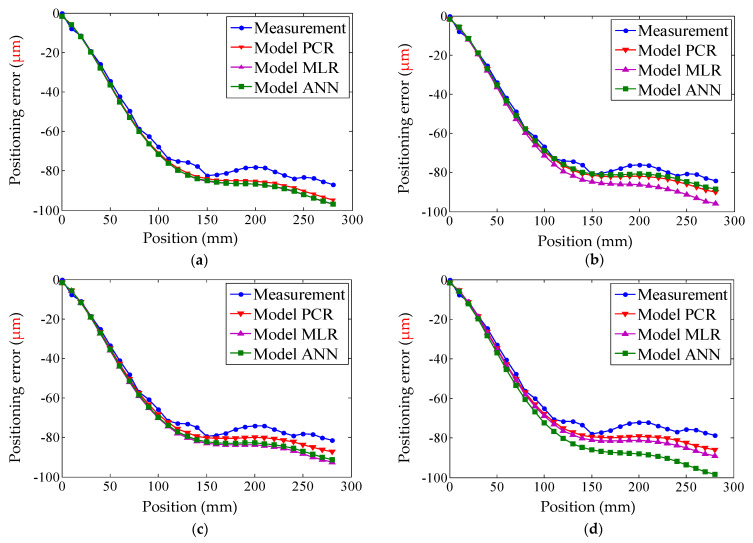
Prediction result of each curve: (**a**) result of the error modeling at 40 min; (**b**) result of the error modeling at 120 min; (**c**) result of the error modeling at 160 min; and (**d**) result of the error modeling at 220 min.

When the machine tool had been warmed up for 40, 120, 160, and 220 min (represented by the numbers 0, 1, 2, and 3), the measured positioning errors were compared with the predicted positioning errors calculated by three positioning error models. The modeling results are shown in Figure 17, Figure 18 and Figure 19, the residual error can be seen in Figure 20, and the details of each positioning error curve can be observed in Figure 21. The performances of three models are evaluated in Table 21. The prediction accuracy of the PCR model can reach about 86%, which is the highest. The prediction accuracy of the MLR model is higher than that of the ANN model.

In experiment I, the fitting accuracy of the PCR model is the lowest. However, in experiment II, the prediction accuracy of the PCR model is in fact the best. The selection method of the temperature-sensitive points in this paper, to some extent, can reduce the multi-collinearity among the input variables. However, when the input variables are selected, what should be first considered is that the fitting accuracy of the error model is the highest. Therefore, multi-collinearity still exists among the selected input variables. Then PCR is applied to establishing the thermal error model which does not have the problem of multi-collinearity. The prediction accuracy of the PCR model is better than that of the other two models, which means that the PCR model is of strong robustness.

If the model had the problem of multi-collinearity, when the conditions of the experiment changed, i.e., the distribution of temperatures was different, the prediction accuracy would degrade. Therefore, the PCR model is more suitable for thermal error modeling.

## 5. Conclusions

Based on empirical modeling and experimental analysis, a modeling method of thermal positioning error is presented in this paper. The experimental results showed that the predicted positioning errors were well matched with the measured positioning errors at any time during the machine tool operation. The following conclusions can be drawn:

(1) The thermal positioning error model is constructed by empirical modeling. The initial geometric positioning error is modeled with polynomial fitting, and the thermal positioning error is predicted by principal component regression. The high-precision and strong robustness of the error model can be achieved.
(2) Fuzzy clustering analysis of the maximal tree method is applied to classifying the data. A criterion which is the combination of the sample determination coefficient and the significance of the regression equation is presented to search for the optimal clustering result. The optimal clustering can be obtained, and the number of temperature sensors can be reduced from 11 to 5.
(3) PCR is applied to thermal positioning error modeling because PCR can greatly reduce the multi-collinearity among the input variables and improve the robustness of the error model. The positioning error model could accurately predict the positioning error under different thermal states. The fitting accuracy of the error modeling could reach about 89%, and the prediction accuracy could reach about 86%. Hence, the PCR model is a candidate for the thermal modeling.
(4) The proposed thermal error model in this article is verified. Based on the presented model, the corresponding development of the actual hardware equipment will be part of future studies.

## Figures and Tables

**Table 1:** The installation locations of temperature sensors.

Sensor	Location
1	*Y* axis motor
2	Column
3	Front end of *Y* axis guideway
4	Bed
5	Back end of *Y* axis guideway
6	Environment around the machine tool
7	Front bearing of *Y* axis
8	Work table
9	Rear bearing of *Y* axis
10	Leading screw nut
11	Environment of the room

**Table 2:** The slope of each trend line.

No.	Curve Slope *k_i_*	△*k*
0	0	0
1	1	1
2	2	2
3	3	3
4	4	4
5	5	5
6	6	6
7	7	7

**Table 3:** Cluster results of maximal tree method.

Threshold Value of *λ*	Cluster Result
[0,0.94943)	$\left\{T_{1},T_{2},T_{3},T_{4},T_{5},T_{6},T_{7},T_{8},T_{9},T_{10},T_{11}\right\}$
[0.94943,0.96927)	$\left\{T_{1},T_{2},T_{3},T_{4},T_{5},T_{6},T_{7},T_{8},T_{9},T_{10}\},\{T_{11}\right\}$
[0.96927,0.97348)	$\left\{T_{1},T_{2},T_{3},T_{4},T_{5},T_{6},T_{8},T_{9},T_{10}\},\{T_{7}\},\{T_{11}\right\}$
[0.97348,0.97891)	$\left\{T_{1},T_{5},T_{6},T_{9},T_{10}\},\{T_{2},T_{3},T_{4},T_{8}\},\{T_{7}\},\{T_{11}\right\}$
[0.97891,0.98226)	$\left\{T_{1}\},\{T_{2},T_{3},T_{4},T_{8}\}\{T_{5},T_{6},T_{9},T_{10}\},\{T_{7}\},\{T_{11}\right\}$
[0.98226,0.98288)	$\left\{T_{1}\},\{T_{2},T_{3},T_{4},T_{8}\},\{T_{5},T_{6}\},\{T_{7}\},\{T_{9},T_{10}\},\{T_{11}\right\}$
[0.98288,0.98366)	$\left\{T_{1}\},\{T_{2},T_{3},T_{4},T_{8}\},\{T_{5}\},\{T_{6}\},\{T_{7}\},\{T_{9},T_{10}\},\{T_{11}\right\}$
[0.98366,0.98747)	$\left\{T_{1}\},\{T_{2}\},\{T_{3},T_{4},T_{8}\},\{T_{5}\},\{T_{6}\},\{T_{7}\},\{T_{9},T_{10}\},\{T_{11}\right\}$
[0.98747,0.98947)	$\left\{T_{1}\},\{T_{2}\},\{T_{3}\},\{T_{4},T_{8}\},\{T_{5}\},\{T_{6}\},\{T_{7}\},\{T_{9},T_{10}\},\{T_{11}\right\}$
[0.98947,0.9921)	$\left\{T_{1}\},\{T_{2}\},\{T_{3}\},\{T_{4},T_{8}\},\{T_{5}\},\{T_{6}\},\{T_{7}\},\{T_{9}\},\{T_{10}\},\{T_{11}\right\}$
[0.9921,1]	$\left\{T_{1}\},\{T_{2}\},\{T_{3}\},\{T_{4}\},\{T_{5}\},\{T_{6}\},\{T_{7}\},\{T_{8}\},\{T_{9}\},\{T_{10}\},\{T_{11}\right\}$

**Table 4:** The correlation coefficients between temperature variables and slope $k_{i}$.

Temperature Variable	Correlation Coefficient	Temperature Variable	Correlation Coefficient
T1	0.9725	T7	0.8536
T2	0.9660	T8	0.9382
T3	0.9538	T9	0.9667
T4	0.9160	T10	0.9440
T5	0.9814	T11	0.8889
T6	0.9673		

**Table 5:** The combination of the representative variables in each cluster.

No.	Cluster Result
1	*T*_5_,
2	*T*_5_,*T*_11_
3	*T*_5_,*T*_7_,*T*_11_
4	*T*_2_,*T*_5_,*T*_7_,*T*_11_
5	*T*_1_,*T*_2_,*T*_5_,*T*_7_,*T*_11_
6	*T*_1_,*T*_2_,*T*_5_,*T*_7_,*T*_9_,*T*_11_
7	*T*_1_,*T*_2_,*T*_5_,*T*_6_,*T*_7_,*T*_9_,*T*_11_
8	*T*_1_,*T*_2_,*T*_3_,*T*_5_,*T*_6_,*T*_7_,*T*_9_,*T*_11_
9	*T*_1_,*T*_2_,*T*_3_,*T*_5_,*T*_6_,*T*_7_,*T*_8_,*T*_9_,*T*_11_
10	*T*_1_,*T*_2_,*T*_3_,*T*_5_,*T*_6_,*T*_7_,*T*_8_,*T*_9_,*T*_10_,*T*_11_
11	*T*_1_,*T*_2_,*T*_3_,*T*_4_,*T*_5_,*T*_6_,*T*_7_,*T*_8_,*T*_9_,*T*_10_,*T*_11_

**Table 6:** Sample determination coefficient *R*^2^.

The Number of Variables	*R* ^2^
1	0.963
2	0.963
3	0.971
4	0.989
5	0.997
6	0.999

**Table 7:** Variance analysis of 5 variables.

Model		Sum of Squares	df	Mean Square	*F*	Sig.
1	Regression	0.012	5	0.002	120.014	0.008
	Residual	0.000	2	0.000		
	Total	0.012	7			

**Table 8:** Variance analysis of 6 variables.

Model		Sum of Squares	df	Mean Square	*F*	Sig.
1	Regression	0.012	6	0.002	170.838	0.058
	Residual	0.000	1	0.000		
	Total	0.012	7			

**Table 9:** Model summary of MLR.

Model	*R*	*R* Square	Adjusted *R* Square	Std. Error of the Estimate
1	0.998	0.997	0.988	0.0044024

**Table 10:** Coefficients of MLR.

Model		Unstandardized Coefficients		Standardized Coefficients	*t*	Sig.
		B	Std. Error	Beta		
1	(Constant)	−0.8782	0.3738		−2.3492	0.1433
	T1	0.0178	0.0081	0.5553	2.1937	0.1595
	T2	0.0946	0.0244	1.0014	3.8770	0.0605
	T5	−0.0016	0.0195	−0.0331	−0.0817	0.9423
	T7	−0.0608	0.0166	−0.4553	−3.6675	0.0670
	T11	−0.0238	0.0235	−0.1006	−1.0133	0.4176

**Table 11:** Total variance explained.

Component	Initial Eigen values			Extraction Sums of Squared Loadings		
	Total	Percentage of Variance	Cumulative Percentage	Total	Percentage of Variance	Cumulative Percentage
1	4.645	92.896	92.896	4.645	92.896	92.896
2	0.194	3.880	96.776			
3	0.118	2.367	99.143			
4	0.036	0.725	99.868			
5	0.007	0.132	100.000			

**Table 12:** Model summary of the PCR.

Model	*R*	*R* Square	Adjusted *R* Square	Std. Error of the Estimate
1	0.968	0.938	0.927	0.0110105

**Table 13:** Variance analysis of PCR.

Model		Sum of Squares	df	Mean Square	*F*	Sig.
1	Regression	0.011	1	0.011	90.326	0.000
	Residual	0.001	6	0.000		
	Total	0.012	7			

**Table 14:** Coefficients of PCR.

Model		Unstandardized Coefficients		Standardized Coefficients	*t*	Sig.
		B	Std. Error	Beta		
1	(Constant)	−0.3243	0.0039		−83.3080	0.0000
	C1	0.0396	0.0042	0.9684	9.5040	0.0000

**Table 15:** Model summary of principal component 1.

Model	*R*	*R* Square	Adjusted *R* Square	Std. Error of the Estimate
1	1.000	1.000	1.000	0.00000000

**Table 16:** Variance analysis principal component 1.

Model		Sum of Squares	df	Mean Square	*F*	Sig.
1	Regression	7.000	5	1.400		
	Residual	0.000	2	0.000		
	Total	7.000	7			

**Table 17:** Coefficients of principal component 1.

Model		Unstandardized Coefficients		Standardized Coefficients	t	Sig.
		B	Std. Error	Beta		
1	(Constant)	−50.6062	0.0000		−51938166.3663	0.0000
	T1	0.1631	0.0000	0.2084	7731564.4627	0.0000
	T2	0.4902	0.0000	0.2120	7708140.8593	0.0000
	T5	0.2512	0.0000	0.2132	4941567.5591	0.0000
	T7	0.6614	0.0000	0.2023	15306040.9534	0.0000
	T11	1.1666	0.0000	0.2015	19050321.8342	0.0000

**Table 18:** MSE of the neural network with different nodes in the hidden layer.

Number of Nodes	MSE
3	1.137 × 10^−5^
4	9.9999 × 10^−6^
5	9.9909 × 10^−6^
6	9.9962 × 10^−6^
7	2.0819 × 10^−5^

**Table 19:** *R*^2^ of the neural network with different nodes in the hidden layer.

Number of Nodes	*R* ^2^
3	0.9961
4	0.9966
5	0.9966
6	0.9966
7	0.9928

**Table 20:** Fitting results of three models.

Model	RMSE	MAXR	MINR
PCR	1.9978	3.9	−6
MLR	1.3134	2.1	−3.4
ANN	1.3357	2.8	−3.3

MAXR means maximal residual error, and MINR means minimum residual error.

**Table 21:** Prediction results of three models.

Model	RMSE	MAXR	MINR
PCR	4.2093	8.3	−2.2
MLR	6.4909	12.1	−2.1
ANN	7.3053	19.7	−2.3
